# Hypometabolic subtypes of AD are linked to comorbid hippocampal sclerosis and Lewy body pathology

**DOI:** 10.1186/s13195-025-01796-6

**Published:** 2025-07-25

**Authors:** Fedor Levin, Martin Dyrba, Stefan J. Teipel, Michel J. Grothe

**Affiliations:** 1 Deutsches Zentrum für Neurodegenerative Erkrankungen (DZNE), Rostock, Germany; 2 Department of Psychosomatic Medicine, Rostock University Medical Center, Rostock, Germany; 3 Reina Sofia Alzheimer Center, CIEN Foundation, ISCIII, Madrid, Spain

## Abstract

**Background:**

Neuroimaging studies have identified distinct ‘typical/neocortical’ and ‘limbic-predominant’ hypometabolic subtypes of AD with different clinical and biomarker characteristics. We investigated associations of these subtypes with postmortem neuropathological measures in an observational study.

**Methods:**

Antemortem FDG-PET scans of 74 participants from the ADNI autopsy cohort were classified into previously described typical/neocortical and limbic-predominant subtype patterns. We used Bayesian regression and ANCOVA to test associations between the subtypes and neuropathological features.

**Results:**

Results were inconclusive for Thal phases, Braak stages, CERAD neuritic plaque scores, hippocampal tangle density, and TDP-43 pathology (BF_10_ between 0.447 and 1.146). However, the limbic-predominant subtype was associated with hippocampal sclerosis (BF_10_ = 3.842, moderate level of evidence), whereas the typical/neocortical subtype was associated with Lewy body pathology (BF_10_ = 10.093, strong level of evidence).

**Conclusions:**

These findings highlight the influence of AD and non-AD-specific pathologies on neurodegeneration patterns and may provide directions for research into hypometabolic pattern analysis as an indirect marker of comorbid pathology.

**Supplementary Information:**

The online version contains supplementary material available at 10.1186/s13195-025-01796-6.

## Introduction

Individuals with Alzheimer’s disease (AD) show considerable clinical and biological heterogeneity, which has led to the characterization of distinct subtypes of AD [1, 2]. Initially, distinct pathologic subtypes of AD were described based on the distributions of cortical and hippocampal neurofibrillary tangles (NFT), resulting in so called ‘typical’, ‘limbic-predominant’, and ‘hippocampal-sparing’ subtypes [3]. Other studies identified topographically similar subtypes based on spatial patterns of gray matter (GM) atrophy or cortical thickness [1]. FDG-PET is a commonly used and more sensitive molecular imaging biomarker of neurodegeneration in AD [4]. Previously, we used a data-driven subtyping analysis of FDG-PET images and identified common temporo-parietal (‘typical/neocortical’) and ‘limbic-predominant’ hypometabolic subtypes of AD, together with a rare ‘cortical-predominant’ subtype with more prominent frontal hypometabolism [5]. The limbic-predominant subtype, which was almost as common as the ‘typical/neocortical’ temporo-parietal subtype, was associated with older age and a more memory-predominant cognitive profile. Similar limbic-predominant hypometabolic subtypes have also been described in other research studies using hypothesis-driven approaches for identifying hypometabolic subtypes, and a relation of this subtype with limbic TDP-43 pathology and hippocampal sclerosis has been hypothesized [6–8]. Limbic TDP-43 pathology has been estimated to be present in approximately 50% of AD cases, and it has been linked to more severe hippocampal atrophy [9, 10]. Moreover, limbic TDP-43 pathology has been classified as a distinct pathologic entity within the concept of limbic age-related TDP-43 encephalopathy neuropathological change (LATE-NC), which is associated with hippocampal sclerosis and can show a similar amnestic phenotype as AD dementia [11, 12]. In a recent imaging-pathologic association study, we found that autopsy cases with LATE-NC were characterized by a distinct ‘temporo-limbic’ FDG-PET pattern that showed some similarity to the limbic-predominant hypometabolic subtype described in AD [11]. On the other hand, comorbid Lewy body (LB) pathology is also estimated to be present in approximately 50% of AD cases [13], and has been shown to influence both the clinical phenotype and the pattern of regional neurodegeneration [14–16], albeit in a different manner compared to limbic TDP-43 [17, 18]. Thus, while limbic TDP-43 pathology has been associated with a more memory-predominant cognitive profile and more pronounced medial temporal lobe atrophy, LB pathology typically associates with relatively more pronounced executive and visuospatial deficits and less pronounced medial temporal involvement. However, the degree to which the recently described ‘typical/neocortical’ and ‘limbic-predominant’ hypometabolic subtypes of AD may be affected by non-AD pathologies, in addition to potential differences in AD pathology distribution, is currently largely unknown [5, 6, 8].

In the current study, we analyzed hypometabolic patterns in antemortem FDG-PET data of participants with postmortem neuropathological examination to evaluate potential relations of the hypometabolic AD subtype patterns with AD- and non-AD-specific neuropathological features.

## Methods

### Participants

We analyzed data from participants in the Alzheimer’s Disease Neuroimaging Initiative (ADNI) autopsy cohort with available postmortem neuropathological assessments (release date 14th of November 2022) and with available antemortem FDG-PET assessments conducted fewer than 10 years before death. To analyze associations of various AD- and non-AD-specific neuropathological features with subtype-related hypometabolic patterns, we aimed to include participants representing a broad continuum with respect to AD-related neuropathology. Therefore, we did not apply additional inclusion criteria with respect to antemortem clinical diagnosis or postmortem evidence of moderate/advanced AD neuropathology. The resulting sample included 74 participants, including 7 with normal cognition (CN), 12 with MCI, and 55 with dementia according to the last clinical examination 2.1 ± 1.7 years before death.

Specific inclusion criteria for different diagnostic groups within the ADNI study are listed on the ADNI website (https://adni.loni.usc.edu/methods/documents/). The ADNI was launched in 2003 as a public-private partnership, led by Principal Investigator Michael W. Weiner, MD. The ADNI is a longitudinal multicenter study aimed at investigating whether neuroimaging methods such as MRI and PET, together with other biological, genetic, clinical and neuropsychological measures can be used to characterize progression of mild cognitive impairment (MCI) and early Alzheimer’s disease (AD). For up-to-date information, see https://adni.loni.usc.edu.

### FDG-PET data preprocessing and analysis

We retrieved the last available FDG-PET scan from each participant to characterize its similarity to the typical/neocortical temporo-parietal and limbic-predominant hypometabolic subtypes of AD. Average delay between FDG-PET scan and death was 3.3 ± 2 years. A detailed description of acquisition and image pre-processing for inter-site harmonization is presented at the ADNI website (https://adni.loni.usc.edu/help-faqs/adni-documentation/). These pre-processed images were normalized to a customized FDG-PET template, smoothed with a Gaussian kernel of 8 mm full-width at half maximum, and intensity-scaled to the global mean signal [5, 19].

The typical/neocortical and limbic-predominant hypometabolic subtypes were identified previously using clustering analysis of identically processed FDG-PET data from 174 Aβ-positive participants with AD dementia as described in our earlier study [20]. Specifically, the subtypes were identified using Ward’s hierarchical clustering performed on individual voxel-wise FDG-PET profiles scaled to the global mean signal [5, 20]. Based on objective clustering diagnostic measures, that previous study identified the typical/neocortical and limbic-predominant subtypes as well as a rare cortical-predominant subtype [5]. However, we did not analyze the cortical-predominant subtype in the current study due to the low number of participants. The autopsy sample used in the present study and the clustering sample used for subtype identification in our previous study had overlapping participants. We excluded these participants from the definition of the averaged subtype templates (9 participants from the typical/neocortical subtype and 9 participants from the limbic-predominant subtype) to avoid a potential circularity in the definition and subsequent characterization of the subtypes. However, additional analyses showed that the exclusion of these participants had no notable effect on the subtype templates and similarity analyses. To measure similarity of individual FDG-PET profiles to the typical/neocortical and the limbic-predominant subtype we used Euclidean distances [5, 20]. We characterized the relative similarity by calculating the ratio of Euclidean distances between the individual voxel-wise FDG-PET profile and the respective subtype templates. Thus, the ratio represents the similarity of an individual FDG-PET profile to the limbic-predominant subtype pattern relative to the typical/neocortical subtype pattern. Values of the ratio higher than 1 indicate that a participant shows higher similarity to the limbic-predominant subtype, whereas values below 1 indicate a higher similarity to the typical/neocortical subtype. To visualize the typical/neocortical (*n* = 75) and the limbic-predominant (*n* = 69) subtype templates, we compared the voxel-wise hypometabolic patterns of the respective subtypes to a cognitively normal control group (*n* = 179) as described previously [5]. Specifically, for this analysis FDG-PET scans were adjusted to the average pons signal, and age, sex, and years of education were included as covariates. For visualization of the regional effect sizes, the voxel-wise maps were converted into Z-scores (Supplementary Fig. 1).

### Neuropathological measures

All neuropathological evaluations in the ADNI cohort are performed through the central laboratory of the ADNI neuropathology core (http://adni.loni.usc.edu/about/#core-container), including a standardized assessment of a wide range of AD and non-AD neuropathological lesions as captured in the Neuropathology Data Form Versions 10 and 11 of the National Alzheimer Coordinating Center (available at https://naccdata.org/data-collection/forms-documentation/np-10 and https://naccdata.org/data-collection/forms-documentation/np-11).

Here, we used established rating scales for AD neuropathologic change represented by Thal amyloid phases, Braak neurofibrillary tangle (NFT) staging and CERAD neuritic plaque scores, as well as ratings of non-AD pathologies including TDP-43 pathology, hippocampal sclerosis, and Lewy body pathology. We operationalized severity of TDP-43 pathology as simple count of brain areas with TDP-43 immunoreactive inclusions (assessed in amygdala, hippocampus, entorhinal/inferior temporal cortex and neocortex). For two participants, data entries for amygdala TDP-43 inclusions were missing and were omitted from the sum. Due to limited available data on hippocampal sclerosis, it was operationalized as a binary variable indicating absence or presence (either unilateral, bilateral, or without assessed laterality). Neuropathological measures of Lewy body pathology were available as categories indicating presence of the pathology across brain regions, including brainstem, limbic region, amygdala, neocortex, and olfactory bulb [21]. Therefore, we operationalized LB pathology as a binary variable indicating absence or presence in any of the assessed brain regions. In addition, we assessed semi-quantitative ratings [0–3] of hippocampal NFT severity (assessed in the CA1 subfield), which were available for a subsample of 49 of participants.

### Biomarkers and neuropsychological test scores

To better characterize the selected sample, we retrieved information on CSF biomarkers, as well as neuropsychological test scores available in ADNI. CSF amyloid, total tau and p-tau measures were obtained using Elecsys cobas e 601^1^ [22], and we selected data that were collected within one year from the analyzed FDG-PET scans for a total of 47 participants. Additionally, we selected data on CSF alpha-synuclein presence evaluated with the alpha-synuclein seed amplification assay (αSyn SAA), which was available for 40 participants [23]. To characterize cognitive performance of participants in the sample, we use MMSE scores, as well as ADNI memory (ADNI-MEM) and executive function (ADNI-EF) composite scores [24, 25].

### Statistical analysis

We analyzed the data using JASP (Jeffreys’s Amazing Statistics Program, version 0.17.1; JASP Team, 2023), which allows conducting analyses using Bayesian statistics. The Bayesian framework offers an advantage over the more conventional null hypothesis significance testing within frequentist statistics in that it allows to quantify evidence for existence or absence of a specific effect as compared to the null hypothesis [26]. Therefore, it can also provide a more straightforward proof of the lack of an effect. For all JASP analyses we primarily used default settings, unless specified otherwise.

As a first step, we used Bayesian linear regressions to investigate relationships of individual subtype similarity with the assessed neuropathological measures, accounting for age, sex, and delay between FDG-PET scan and death as covariates. As a criterion for judging the statistical association, we used Bayes factors (BF), such as BF_10_, which indicates the relative evidence for a given model in comparison to a null model. Following established standards in the field, we interpreted BF_10_ < 0.33 as indicating moderate evidence for the null hypothesis, and BF_10_ > 3 and BF_10_ > 10 as indicating moderate and strong evidence, respectively, for the alternative hypothesis [26, 27]. We interpreted BF_10_ values between 0.33 and 3 corresponding to anecdotal level of evidence as inconclusive. In the outputs from model comparisons, we also report BF_M_– BF representing informativeness of the data given prior and posterior distributions, as well as P(M) and P(M|data)– prior and posterior distributions, respectively. Additionally, we report posterior summaries of coefficients with estimates of parameters and 95% credible intervals. We conducted an additional test evaluating a different operationalization of the TDP-43 pathology measure as a binary variable. In a further exploratory test, we also conducted Bayesian regression analysis in a subsample of participants with ADNC scores of 1 and higher (*n* = 70).

In a complementary analysis, we conducted Bayesian ANCOVA comparing subtype similarity across four pathologic groups of interest, which were defined by the presence/absence of AD and TDP-43 pathology. Presence of pathologic AD required at least moderate levels of AD neuropathologic change (i.e., ADNC ≥ 2) [28]. This resulted in four groups characterized by (i) neither AD nor TDP-43 pathology, (ii) primarily AD pathology without TDP-43, (iii) primarily TDP-43 pathology without AD, and (iv) both AD and TDP-43 pathology. Age, sex, and delay between FDG-PET scan and death were included as covariates. Post-hoc t-tests were performed to compare groups directly. Additionally, we conducted analogous Bayesian ANCOVA across four groups defined by the presence or absence of AD and LB pathology as: (i) neither AD nor LB pathology, (ii) primarily AD pathology without LB, (iii) primarily LB pathology without AD, and (iv) both AD and LB pathology. Finally, for a more detailed evaluation of different categories within the LB variable, we conducted additional analyses comparing a group of participants with limbic or amygdala-predominant LB to a group without LB pathology; and comparing a group of participants with neocortical LB to a group without LB pathology.

## Results

Characteristics of participants from the full autopsy sample, as well as subgroups stratified by AD and TDP-43 neuropathology, are presented in Table 1. In addition to that, we also present characteristics of participants stratified by AD and LB neuropathology in Supplementary Table 1. The average time interval between the last FDG-PET assessment and death was 3.3 years, with a large majority of participants (86%) having a delay of 5 years or fewer. The sample included 54 participants with ADNC score of 2 or 3, as well as 16 participants with ADNC score of 1 representing early AD neuropathology, and four participants with ADNC score of 0.

**Table 1:** Demographic, neuropathological, biomarker and clinical characteristics of the autopsy cohort

	Full autopsy cohort	Neither AD nor TDP-43 pathology	AD pathology only	TDP-43 pathology only	AD and TDP-43 pathology
Demographics					
n (%)	74 (100%)	11 (15%)	29 (39%)	9 (12%)	25 (34%)
CN/MCI/AD	7/12/55	5/2/4	1/4/24	0/3/6	1/3/21
Age at death, years	79.61 (7.24)	81.65 (6.86)	78.37 (7.77)	82.42 (4.57)	79.13 (7.45)
Sex, female, n (%)	19 (26%)	3 (27%)	8 (28%)	1 (11%)	7 (28%)
Education, years	16.27 (2.79)	16.91 (3.3)	16.24 (2.52)	15.78 (2.95)	16.2 (2.93)
Delay from FDG-PET to death, years	3.31 (2.01)	3 (1.34)	3.03 (1.78)	3.11 (2.89)	3.84 (2.13)
Biomarkers and neuropathological measures					
APOE ε4, n (%)	40 (54%)	1 (9%)	22 (76%)	1 (11%)	16 (64%)
Hippocampal sclerosis, n (%)	6 (8%)	0 (0%)	0 (0%)	3 (33%)	3 (12%)
Braak stages (0/1/2/3/4/5/6)	1/6/11/2/4/38/12	1/2/7/0/0/1/0	0/0/0/2/1/19/7	0/4/4/0/1/0/0	0/0/0/0/2/18/5
Thal phases (0/1/2/3/4/5)	4/5/1/8/18/38	3/2/1/1/2/2/	0/0/0/2/6/21	1/3/0/5/0/0	0/0/0/0/10/15
CERAD neuritic plaque score (0/1/2/3)	17/9/8/40	10/1/0/0	2/2/4/21	5/4/0/0	0/2/4/19
ADNC (0/1/2/3)	4/16/6/48	3/8/0/0	0/0/4/25	1/8/0/0	0/0/2/23
TDP-43 severity	1.26 (1.54)	0	0	3.22 (0.83)	2.56 (1.08)
CA1 NFT density	2.35 (0.95)	1.13 (0.64)	2.7 (0.66)	1.5 (1.22)	2.87 (0.35)
Lewy body pathology, n (%)	41 (55%)	5 (45%)	14 (48%)	5 (56%)	17 (68%)
Lewy body pathology categories (0/1/2/3/4/5)	33/4/6/18/10/3	6/0/1/3/0/1/	15/2/1/5/6/0	4/2/1/2/0/0	8/0/3/8/4/2
CSF Aβ, pg/ml	934.8 (843.39)	2070.15 (1244.13)	660.87 (310.24)	1904 (1034.48)	542.59 (224.77)
CSF t-tau, pg/ml	335.78 (128.23)	272.9 (116.52)	358.49 (128.24)	252.67 (89.47)	354.87 (132.29)
CSF p-tau, pg/ml	31.79 (14.4)	22.26 (7.35)	35.72 (15.17)	19.34 (7)	34.16 (14.52)
CSF αSyn SAA, n (%)	19 (48%)	2 (33%)	5 (33%)	2 (67%)	10 (62%)
Cognition					
MMSE	20.14 (6.67)	26.27 (3.58)	18.76 (6.33)	24.11 (3.06)	17.6 (6.92)
ADNI-MEM	-0.91 (1.1)	0.39 (1.27)	-1.07 (1.06)	-0.63 (0.52)	-1.35 (0.8)
ADNI-EF	-1.06 (1.31)	-0.49 (1.52)	-1.37 (1.32)	-0.25 (1.21)	-1.26 (1.09)
Subtyping characteristics					
Euclidean distance to the limbic-predominant subtype	38.51 (9.58)	40.67 (9.35)	38.29 (10.95)	35.61 (8.07)	38.88 (8.7)
Euclidean distance to the typical/neocortical subtype	38.67 (7.67)	37.59 (5.47)	39.21 (8.57)	41.98 (7.43)	37.34 (7.46)
Similarity to the limbic-predominant relative to the typical/neocortical subtype	1.03 (0.19)	0.95 (0.16)	1.06 (0.19)	1.2 (0.19)	0.97 (0.14)
Number of participants with higher similarity to the limbic-predominant subtype	43 (58%)	5 (45%)	19 (66%)	8 (89%)	11 (44%)

Sample sizes are presented with percentages relative to the respective group in parentheses. AD pathology is defined as ADNC score ≥ 2. TDP-43 pathology is defined as any TDP-43 inclusions identified in the assessed brain regions. Participants with higher similarity to the limbic-predominant subtype are defined as participants with values of similarity to the limbic-predominant relative to the typical/neocortical subtype higher than 1. Values for variables are presented as numbers of corresponding participants with percentages in parentheses (for sex, APOE ε4 genotype, hippocampal sclerosis, Lewy body pathology, number of participants with higher similarity to the limbic-predominant subtype), or means with standard deviation in parentheses, or simple counts of values (diagnostic group, Braak stages, Thal phases, CERAD neuritic plaque score, ADNC score, Lewy body pathology categories). Lewy body pathology categories: 0 = none, 1 = brainstem predominant, 2 = limbic (transitional), 3 = neocortical (diffuse), 4 = amygdala-predominant, 5 = olfactory bulb. CSF αSyn SAA represents number of participants positive for LB pathology as assessed via the α-synuclein seed amplification assay. Missing values are excluded

### Subtype similarity and neuropathological measures in regression analyses

**Fig. 1:**
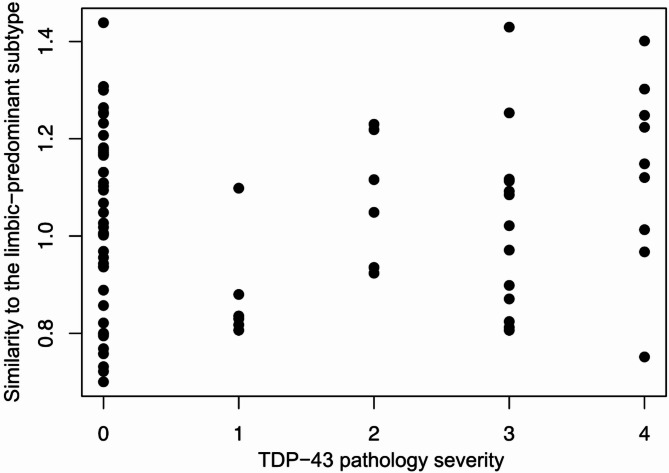
TDP-43 pathology severity and similarity to the limbic-predominant hypometabolic subtype. TDP-43 pathology severity is represented by the number of brain areas with TDP-43 immunoreactive inclusions, as assessed in amygdala, hippocampus, entorhinal/inferior temporal cortex and neocortex. Similarity to the limbic-predominant subtype is represented by the ratio of Euclidean distance to the average typical/neocortical subtype profile and Euclidean distance to the average limbic-predominant subtype profile

In Bayesian linear regressions, evidence for an association between subtype similarity and neuropathological features was inconclusive for Thal phases (BF_10_ = 0.757), Braak stages (BF_10_ = 1.146), CERAD neuritic plaque scores (BF_10_ = 0.680), and severity of TDP-43 pathology (BF_10_ = 0.744) (Fig. 1; Table 2, Supplementary Table 2 for posterior summary of coefficients). However, results supported an association of hippocampal sclerosis with higher similarity to the limbic-predominant subtype at a moderate level of evidence (BF_10_ = 3.842). In addition, Lewy body pathology was associated with a higher similarity to the typical/neocortical subtype with a strong level of evidence (BF_10_ = 10.093). An additional test with the TDP-43 pathology measure operationalized as a binary variable also yielded inconclusive evidence (BF_10_ = 0.430, see Supplementary Tables 3 and 4). Regression analysis in a subsample of participants with available regional pathologic measures (*n* = 49) suggested inconclusive evidence for an association between hippocampal CA1 NFT density and the similarity to the limbic-predominant subtype (BF_10_ = 0.447; Table 3, Supplementary Table 5 for posterior summary of coefficients). Exploratory analysis in a subsample of participants with ADNC scores of 1 and higher showed results consistent with our main results (Supplementary Tables 6 and 7).

**Table 2:** Bayesian regression analysis– comparison of models predicting similarity to the limbic-predominant subtype

	*P*(M)	*P*(M|Data)	BF_M_	BF_10_	*R* ^2^
Null model (including age, sex, interval FDG-PET to death)	0.143	0.055	0.347	1.000	0.104
Thal phase	0.024	0.007	0.285	0.757	0.123
Braak stage	0.024	0.010	0.433	1.146	0.136
TDP-43 pathology	0.024	0.007	0.280	0.744	0.122
Hippocampal sclerosis	0.024	0.035	1.488	3.842	0.172
Lewy body pathology	0.024	0.092	4.155	10.093	0.199
CERAD neuritic plaque score	0.024	0.006	0.256	0.680	0.120

Rows with variables represent separate models in which only the specified variable is included; each model is compared to the null model with age, sex and interval between FDG-PET and death. P(M)– prior distribution, P(M|Data)– posterior distribution, BF_M_ represents how informative data is given prior and posterior distributions, BF_10_– BF in favor of the respective model vs. the null model

**Table 3:** Bayesian regression analysis– comparison of models predicting similarity to the limbic-predominant subtype in a subsample (*n* = 49)

	*P*(M)	*P*(M|Data)	BF_M_	BF_10_	*R* ^2^
Null model (including age, sex, interval FDG-PET to death)	0.500	0.691	2.237	1.000	0.161
CA1 NFT density	0.500	0.309	0.447	0.447	0.161

Model is compared to the null model with age, sex and interval between FDG-PET and death. P(M)– prior distribution, P(M|Data)– posterior distribution, BF_M_ represents how informative data is given prior and posterior distributions, BF_10_– BF in favor of the respective model vs. the null model

### Comparisons of subtype similarity between pathologic groups of interest

Out of the 74 study participants, 11 had neither pronounced AD pathology nor TDP-43, 29 had AD pathology only, 9 had evidence of TDP-43 pathology only, and 25 had both (AD + TDP-43). Bayesian ANCOVA showed differences between these groups with respect to subtype similarity (Table 4; BF_10_ = 10.841). Post-hoc tests showed that the group with only TDP-43 pathology had a higher similarity to the limbic-predominant subtype as compared to all other groups (BF_10_ ranging from 1.577 to 46.444; Table 5 and Supplementary Table 8). Interestingly, the AD + TDP-43 group did not show higher similarity to the limbic-predominant subtype compared to the AD only group (BF_10_ = 0.992). We visualized the distribution of the subtype similarity measure in these groups divided by AD and TDP-43 pathology (Supplementary Fig. 2), as well as groups divided by last available diagnosis (Supplementary Fig. 3) and presence of hippocampal sclerosis or LB pathology (Supplementary Fig. 4).

**Table 4:** Bayesian ANCOVA– comparison of models predicting similarity to the limbic-predominant subtype by pathology groups split by AD and TDP-43 pathology

	*P*(M)	*P*(M|Data)	BF_M_	BF_10_	Error %
Null model (including age, sex, interval FDG-PET to death)	0.500	0.084	0.092	1.000	
Pathology group	0.500	0.916	10.841	10.841	0.959

P(M)– prior distribution, P(M|Data)– posterior distribution, BF_M_ represents how informative data is given prior and posterior distributions, BF_10_– BF in favor of the respective model vs. the null model. Error % represents numerical error of BF

**Table 5:** Bayesian ANCOVA– post-hoc t-tests in the analysis comparing similarity to the limbic-predominant subtype across pathology groups split by AD and TDP-43 pathology

Group 1	Group 2	Prior Odds	Posterior Odds	BF_10,_ uncorrected	Error %
No AD, no TDP-43	AD only	0.414	0.390	0.942	0.004
No AD, no TDP-43	TDP-43 only	0.414	4.060	9.802	0.000
No AD, no TDP-43	AD and TDP-43	0.414	0.154	0.372	0.003
AD only	TDP-43 only	0.414	0.653	1.577	0.003
AD only	AD and TDP-43	0.414	0.411	0.992	0.008
TDP-43 only	AD and TDP-43	0.414	19.238	46.444	0.000

BF_10_, uncorrected– BF in favor of the alternative hypothesis that values differ. Error % represents numerical error of BF

An additional evaluation of groups of participants divided by presence of AD and LB pathology demonstrated only anecdotal level of evidence for the effect of the group variable on subtype similarity (Supplementary Tables 9–11). In post-hoc tests, there was evidence only for a higher similarity of the group with AD and LB pathology to the typical/neocortical subtype as compared to the group without AD or LB pathology. Across further exploratory analyses evaluating more specifically participants with limbic or amygdala-predominant LB, as well as participants with neocortical LB pathology, we observed evidence that participants with neocortical LB showed a higher similarity to the typical/neocortical subtype, but for participants with limbic or amygdala-predominant LB evidence was inconclusive (Supplementary Tables 12–15).

## Discussion

In the present study, we evaluated associations between antemortem hypometabolic subtypes of AD and postmortem neuropathological measures of AD-specific and non-AD-specific pathology. The key findings from Bayesian regression models suggested that the limbic-predominant hypometabolic subtype is associated with hippocampal sclerosis, whereas the typical/neocortical subtype is associated with Lewy body pathology. By contrast, we did not find evidence for an association of hypometabolic subtype with AD-specific markers, including Thal phases, Braak stages, CERAD neuritic plaque scores, and hippocampal-CA1 NFT density. Interestingly, TDP-43 pathology was not found to be generally associated with hypometabolic subtype neither. However, in a complementary analysis we observed that participants with primarily TDP-43 pathology (i.e., with no or only low levels of AD pathology), but not those with mixed AD + TDP-43 pathology, showed higher similarity to the limbic-predominant hypometabolic subtype than other participants.

These results provide partial support for previously hypothesized associations of hypometabolic subtypes with specific co-pathologies in AD [6–8]. In contrast to our expectations, we did not observe an association of the limbic-predominant hypometabolic subtype with higher hippocampal CA1 NFT burden. However, this analysis was possibly limited by ceiling effects in the employed semi-quantitative ratings, where a majority of participants (63%) had a maximal rating of 3. Moreover, we did not observe a general link between TDP-43 pathology and higher similarity to the limbic-predominant subtype pattern. However, hippocampal sclerosis, which is most often associated with TDP-43 pathology, showed such a link. One possibility is that hippocampal sclerosis is a better predictor of the limbic-predominant hypometabolic pattern than TDP-43 alone because it represents a more advanced stage of TDP-43-related pathology [12]. Imaging-pathological association studies using antemortem structural MRI data have demonstrated an effect of comorbid TDP-43 pathology on more pronounced medial temporal lobe atrophy in AD [10, 29]. In our present study, the lack of a general effect of TDP-43 on hypometabolic subtype is further substantiated by a complementary group comparison showing no evidence for subtype differences between pathology-confirmed AD cases with (AD + TDP-43) and without TDP-43 pathology. This is also in agreement with our previous study showing little differences in brain-wide voxel-wise hypometabolism patterns between these AD + TDP-43 and AD only groups, both showing a fairly typical temporo-parietal pattern of hypometabolism [11]. However, interestingly, a comparably small subgroup of participants with primarily TDP-43 pathology did show a remarkably high similarity to the limbic-predominant hypometabolic subtype. We have previously described that this group is characterized by a specific ‘temporo-limbic’ hypometabolic pattern that clearly differs from the typical temporo-parietal pattern in AD and provides diagnostic utility for in-vivo differential diagnosis between LATE-NC and AD [11]. While this pattern is not identical to the limbic-predominant subtype pattern analyzed in the current study, it does show spatial similarity in its predominance on medial temporal brain regions. This spatial similarity likely explains the enrichment of the TDP-43 only group for the limbic-predominant subtype in our current study, and further indicates that FDG-PET-based differential diagnosis between LATE-NC and limbic-predominant AD may be more difficult compared to typical AD.

Another interesting finding of our study was the link between Lewy body pathology and a higher similarity to the typical/neocortical subtype. This finding is consistent with previous MRI and FDG-PET studies reporting relatively less pronounced medial temporal and more pronounced (posterior) cortical involvement in AD cases with comorbid Lewy body pathology [14, 15, 30]. However, previous findings of comorbid Lewy body pathology across neuropathologically defined AD subtypes have provided mixed results. In studies conducted in two different autopsy cohorts from the Mayo clinic, comorbid Lewy body pathology showed a similar prevalence of approximately 30% in both typical and limbic-predominant subtypes [3, 9]. In a study by Janocko et al. the prevalence of Lewy body pathology was also similar between the typical and the limbic-predominant subtypes [31]. However, excluding amygdala-predominant Lewy bodies from this tally would result in frequencies of Lewy body pathology of 38% in the typical subtype and 16% in the limbic-predominant subtype. Therefore, different regional distributions of Lewy body pathology could be differentially linked to specific AD subtypes [31]. Accordingly, analyses of regional neuropathologic data in the MRI subtyping study by Mohanty and colleagues [29] suggested a specific association between Lewy body pathology in the parahippocampal gyrus and the limbic-predominant atrophy subtype. More research is necessary to better understand the differential spatial distributions of comorbid Lewy body deposition and their effect on regional neurodegeneration patterns in AD [32].

One inherent limitation of the current analysis is the need to use autopsy-derived measures for the assessed pathologies, which necessarily results in considerable time delays between antemortem FDG-PET and neuropathological examinations. This limitation could be avoided in the future if validated in-vivo molecular biomarkers for the respective pathologies become available. Such methods would significantly enhance research on the effects of non-AD-specific pathologies on phenotypic heterogeneity in AD. Although the sample size of our study was comparably large for an imaging-pathologic association study with antemortem FDG-PET data [33], some of the pathologic subgroups were still relatively small. For example, there were only 6 participants with hippocampal sclerosis and only 9 participants in the subgroup which had TDP-43 pathology and no or only low AD pathology. Consequently, relevant results may be hard to replicate, and our findings should be interpreted in this context.

An additional limitation of our study is related to the available measures of neuropathological characteristics. For some variables such as TDP-43 or Lewy body pathology there was only limited information available about the regional distributions of neuropathological features. As a result, we could not use a previously described staging scheme for TDP-43 [12], and opted for using a binarized measure of Lewy body pathology.

The current study used measures of similarity to subtype templates defined originally in participants with biomarker-confirmed AD dementia. Some of the participants included in the analysis did not have a clinical dementia diagnosis at the last available antemortem assessment, or a postmortem confirmation of AD neuropathology (ADNC scores of 2 or 3) which could limit the interpretation of the results. However, including these participants was consistent with our goal of testing the effects of AD-related change on hypometabolism patterns, as well as demonstrating the effect of TDP-43 presence at low levels of AD pathology. Although our past studies on hypometabolic subtypes selected amyloid-positive participants [5, 20], it is unlikely that the inclusion of participants with ADNC scores of 0 has adversely influenced the current results (Supplementary Tables 6 and 7).

Another potential limitation of the current analysis is linked to the known issues with the generalizability of the ADNI cohort. It has been reported that the ethnocultural and educational compositions of the ADNI cohorts up to and including ADNI3 are not representative of the general population of the USA [34]. Generalizability to populations of other countries may also be limited. Finally, there were limitations in the available regional measures for hippocampal (CA1) NFT density, which was only available for a subset of participants, and was based on a semi-quantitative rating with a possible ceiling effect. This limitation is potentially relevant for the lack of a detected relationship between the limbic-predominant hypometabolic subtype and higher hippocampal NFT density.

In this imaging-pathologic association study we explored potential neuropathological features associated with distinct hypometabolic subtypes of AD, and found that presence of hippocampal sclerosis, but not presence of TDP-43 itself, was associated with a higher similarity to the limbic-predominant subtype. Presence of Lewy body pathology was associated with higher similarity to the typical temporo-parietal neocortical subtype. These findings highlight the influence of common non-AD-specific pathologies on regional hypometabolism patterns and may provide directions for further research into hypometabolic pattern analysis as an indirect surrogate marker of non-AD-specific pathologies.

## Electronic supplementary material

Below is the link to the electronic supplementary material.

Supplementary Material 1

## Data Availability

Data analyzed in this study were acquired from the Alzheimer’s Disease Neuroimaging Initiative (ADNI) database (http://adni.loni.usc.edu). ADNI data are shared in a de-identified form and without embargo subject to a review of a data use application by the ADNI Data Sharing and Publications Committee. For further information please refer to the ADNI website (https://adni.loni.usc.edu/data-samples/adni-data/).

## Footnotes

1 The Elecsys β-Amyloid(1–42) CSF immunoassay in use is not a commercially available IVD assay. It is an assay that was under development and for investigational use only. The measuring range of the assay is 200 (lower technical limit)– 1700 pg/mL (upper technical limit). The performance of the assay beyond the upper technical limit was not formally established. Therefore, use of values above the upper technical limit, which are provided based on an extrapolation of the calibration curve, is restricted to exploratory research purposes and is excluded for clinical decision making or for the derivation of medical decision points.

## Abbreviations

AD: Alzheimer’s disease
ADNC: Alzheimer’s disease neuropathologic change
ADNI: Alzheimer’s Disease Neuroimaging Initiative
Aβ: Beta-amyloid
ANCOVA: Analysis of covariance
APOE ε4: Apolipoprotein E epsilon 4 allele
BF: Bayes factor
CN: Cognitively normal
FDG: Fluorodeoxyglucose
LATE-NC: Limbic-predominant age-related TDP-43 encephalopathy neuropathologic change
MCI: Mild cognitive impairment
MMSE: Mini Mental State Examination
MRI: Magnetic resonance imaging
NFT: Neurofibrillary tangles
PET: Positron emission tomography
TDP-43: TAR DNA-binding protein 43
