# Supplementary material for: Hypometabolic subtypes of AD are linked to comorbid hippocampal sclerosis and Lewy body pathology
Source: Alzheimers Res Ther. 2025 Jul 25;17:172. doi: 10.1186/s13195-025-01796-6 (PMC12291356; doi:10.1186/s13195-025-01796-6)
Supplement: Supplementary file 1 — Supplementary Material 1 [file 13195_2025_1796_MOESM1_ESM.docx]

**Supplementary information**

**Hypometabolic subtypes of AD are linked to comorbid hippocampal sclerosis and Lewy body pathology**

Fedor Levin^1^; Martin Dyrba^1^; Stefan J. Teipel^1,2^; Michel J. Grothe^3^

1. Deutsches Zentrum für Neurodegenerative Erkrankungen (DZNE), Gehlsheimer Str. 20, 18147 Rostock, Germany.

2. Department of Psychosomatic Medicine, Rostock University Medical Center, Gehlsheimer Str. 20, 18147 Rostock, Germany.

3. Reina Sofia Alzheimer Center, CIEN Foundation, ISCIII, C. de Valderrebollo, 5, Vallecas, 28031 Madrid, Spain.

Corresponding Authors:

Fedor Levin

DZNE-Site Rostock/Greifswald

Gehlsheimer Str. 20

18147 Rostock, Germany

Email: fedor.levin@dzne.de

Phone: +49-381-4949228

Fax: +49-381-4949472

Michel J. Grothe

Fundación CIEN, Centro Alzheimer Reina Sofía,

C. de Valderrebollo, 5, Vallecas, 28031 Madrid, Spain;

E-mail: mgrothe@fundacioncien.es

Phone: +34 913 85 23 00

**Supplementary table 1**

Demographic, neuropathological, biomarker and clinical characteristics of the autopsy cohort, split into subgroups by AD and LB pathology.

|  | Full autopsy cohort | Neither AD nor LB pathology | AD pathology only | LB pathology only | AD and LB pathology |
| --- | --- | --- | --- | --- | --- |
| Demographics |  |  |  |  |  |
| n (%) | 74 (100%) | 10 (14%) | 23 (31%) | 10 (14%) | 31 (42%) |
| CN/MCI/AD | 7/12/55 | 4/3/3 | 2/4/17 | 1/2/7 | 0/3/28 |
| Age at death, years | 79.61 (7.24) | 80.13 (6.47) | 79.48 (6.99) | 83.87 (4.64) | 78.16 (8.02) |
| Sex, female, n (%) | 19 (26%) | 3 (30%) | 10 (43%) | 1 (10%) | 5 (16%) |
| Education, years | 16.27 (2.79) | 17.6 (2.32) | 16.17 (2.5) | 15.2 (3.46) | 16.26 (2.86) |
| Delay from FDG-PET to death, years | 3.31 (2.01) | 2.9 (1.85) | 3.57 (1.83) | 3.2 (2.44) | 3.29 (2.1) |
| Biomarkers and neuropathological measures |  |  |  |  |  |
| APOE ε4, n (%) | 40 (54%) | 2 (20%) | 12 (52%) | 0 | 26 (84%) |
| Hippocampal sclerosis, n (%) | 6 (8%) | 1 (10%) | 3 (13%) | 2 (20%) | 0 |
| Braak stages (0/1/2/3/4/5/6) | 1/6/11/2/4/38/12 | 1/5/3/0/0/1/0 | 0/0/0/2/1/15/5 | 0/1/8/0/1/0/0 | 0/0/0/0/2/22/7 |
| Thal phases (0/1/2/3/4/5) | 4/5/1/8/18/38 | 3/2/1/3/0/1 | 0/0/0/2/7/14 | 1/3/0/3/2/1 | 0/0/0/0/9/22 |
| CERAD neuritic plaque score (0/1/2/3) | 17/9/8/40 | 8/2/0/0 | 2/1/2/18 | 7/3/0/0 | 0/3/6/22 |
| ADNC (0/1/2/3) | 4/16/6/48 | 3/7/0/0 | 0/0/3/20 | 1/9/0/0 | 0/0/3/28 |
| TDP-43 severity | 1.26 (1.54) | 1.4 (1.84) | 1.13 (1.69) | 1.5 (1.72) | 1.23 (1.33) |
| CA1 NFT density | 2.35 (0.95) | 1 (1.1) | 2.73 (0.59) | 1.5 (0.76) | 2.8 (0.52) |
| Lewy body pathology, n (%) | 41 (55%) | 0 | 0 | 10 (100%) | 31 (100%) |
| Lewy body pathology categories (0/1/2/3/4/5) | 33/4/6/18/10/3 | 10/0/0/0/0/0 | 23/0/0/0/0/0 | 0/2/2/5/0/1 | 0/2/4/13/10/2 |
| CSF Aβ, pg/ml | 934.8 (843.39) | 2346.17 (1001.05) | 676.21 (313.55) | 1639.24 (1290.55) | 535.09 (219.76) |
| CSF t-tau, pg/ml | 335.78 (128.23) | 252.88 (99.74) | 342.25 (115.43) | 284.78 (121.89) | 369.59 (140.89) |
| CSF p-tau, pg/ml | 31.79 (14.4) | 21.03 (8.43) | 33.07 (13.88) | 21.99 (5.85) | 36.61 (15.5) |
| CSF αSyn SAA, n (%) | 19 (48%) | 0 | 0 | 4 (100%) | 15 (94%) |
| Cognition |  |  |  |  |  |
| MMSE | 20.14 (6.67) | 27.9 (1.97) | 19.04 (7.9) | 22.7 (2.5) | 17.61 (5.44) |
| ADNI-MEM | -0.91 (1.1) | 0.51 (1.15) | -0.84 (1.13) | -0.63 (0.74) | -1.45 (0.72) |
| ADNI-EF | -1.06 (1.31) | 0.34 (1.05) | -1.06 (1.47) | -1.09 (1.25) | -1.51 (0.96) |
| Subtyping characteristics |  |  |  |  |  |
| Euclidean distance to the limbic-predominant subtype | 38.51 (9.58) | 34.83 (6.95) | 37.01 (11.63) | 41.95 (9.63) | 39.71 (8.38) |
| Euclidean distance to the typical/neocortical subtype | 38.67 (7.67) | 40.09 (7.91) | 38.19 (8.95) | 39.05 (5.46) | 38.46 (7.47) |
| Similarity to the limbic-predominant relative to the typical/neocortical subtype | 1.03 (0.19) | 1.16 (0.17) | 1.07 (0.19) | 0.96 (0.21) | 0.98 (0.15) |
| Number of participants with higher similarity to the limbic-predominant subtype | 43 (58%) | 9 (90%) | 15 (65%) | 4 (40%) | 15 (48%) |

Sample sizes are presented with percentages relative to the respective group in parentheses. AD pathology is defined as ADNC score ≥ 2. LB pathology is defined as any LB inclusions identified in the assessed brain regions. Participants with higher similarity to the limbic-predominant subtype are defined as participants with values of similarity to the limbic-predominant relative to the typical/neocortical subtype higher than 1. Values for variables are presented as numbers of corresponding participants with percentages in parentheses (for sex, APOE ε4 genotype, hippocampal sclerosis, Lewy body pathology, number of participants with higher similarity to the limbic-predominant subtype), or means with standard deviation in parentheses, or simple counts of values (diagnostic group, Braak stages, Thal phases, CERAD neuritic plaque score, ADNC score, Lewy body pathology categories). Lewy body pathology categories: 0=none, 1=brainstem predominant, 2=limbic (transitional), 3=neocortical (diffuse), 4=amygdala-predominant, 5=olfactory bulb. CSF αSyn SAA represents number of participants positive for LB pathology as assessed via the α-synuclein seed amplification assay. Missing values are excluded.

**Supplementary table 2.**

Bayesian regression analysis – posterior summaries of coefficients in the models predicting similarity to the limbic-predominant subtype.

| Coefficient | P(incl) | P(excl) | P(incl\|data) | P(excl\|data) | BF_inclusion_ | Mean | SD | 95% Credible Interval | |
| --- | --- | --- | --- | --- | --- | --- | --- | --- | --- |
|  |  |  |  |  |  |  |  | Lower | Upper |
| Intercept | 1.000 | 0.000 | 1.000 | 0.000 | 1.000 | 1.031 | 0.020 | 0.993 | 1.071 |
| Age | 1.000 | 0.000 | 1.000 | 0.000 | 1.000 | 0.004 | 0.003 | -0.002 | 0.009 |
| Sex | 1.000 | 0.000 | 1.000 | 0.000 | 1.000 | 0.015 | 0.044 | -0.068 | 0.105 |
| Interval FDG-PET to death | 1.000 | 0.000 | 1.000 | 0.000 | 1.000 | -0.014 | 0.009 | -0.031 | 0.004 |
| Thal phase | 0.500 | 0.500 | 0.356 | 0.644 | 0.553 | -0.001 | 0.010 | -0.026 | 0.021 |
| Braak stage | 0.500 | 0.500 | 0.395 | 0.605 | 0.652 | -0.004 | 0.012 | -0.033 | 0.017 |
| TDP-43 pathology | 0.500 | 0.500 | 0.389 | 0.611 | 0.636 | 0.003 | 0.009 | -0.013 | 0.026 |
| Hippocampal sclerosis | 0.500 | 0.500 | 0.610 | 0.390 | 1.562 | 0.070 | 0.078 | -0.006 | 0.224 |
| Lewy body pathology | 0.500 | 0.500 | 0.793 | 0.207 | 3.841 | -0.068 | 0.049 | -0.151 | 0.000 |
| CERAD neuritic plaque score | 0.500 | 0.500 | 0.371 | 0.629 | 0.589 | -0.003 | 0.015 | -0.037 | 0.033 |

P(incl) – prior probability of inclusion. P(excl) – prior probability of exclusion. P(incl|data) – posterior probability of inclusion. P(excl|data) – posterior probability of exclusion. BF_inclusion_ – BF in favor of including the effect.

**Supplementary table 3.**

Bayesian regression analysis – comparison of models predicting similarity to the limbic-predominant subtype using a binarized measure of TDP-43 pathology.

|  | P(M) | P(M\|Data) | BF_M_ | BF_10_ | R^2^ |
| --- | --- | --- | --- | --- | --- |
| Null model (including age, sex, interval FDG-PET to death) | 0.500 | 0.699 | 2.325 | 1.000 | 0.104 |
| TDP-43 pathology, binarized | 0.500 | 0.301 | 0.430 | 0.430 | 0.105 |

Model is compared to the null model with age, sex and interval between FDG-PET and death. P(M) – prior distribution, P(M|Data) – posterior distribution, BFM represents how informative data is given prior and posterior distributions, BF10 – BF in favor of the respective model vs the null model.

**Supplementary table 4.**

Bayesian regression analysis – posterior summaries of coefficients in the models predicting similarity to the limbic-predominant subtype using a binarized measure of TDP-43 pathology.

| Coefficient | P(incl) | P(excl) | P(incl\|data) | P(excl\|data) | BF_inclusion_ | Mean | SD | 95% Credible Interval | |
| --- | --- | --- | --- | --- | --- | --- | --- | --- | --- |
|  |  |  |  |  |  |  |  | Lower | Upper |
| Intercept | 1.000 | 0.000 | 1.000 | 0.000 | 1.000 | 1.031 | 0.021 | 0.991 | 1.071 |
| Age | 1.000 | 0.000 | 1.000 | 0.000 | 1.000 | 0.005 | 0.003 | 0.000 | 0.010 |
| Sex | 1.000 | 0.000 | 1.000 | 0.000 | 1.000 | 0.032 | 0.043 | -0.051 | 0.113 |
| Interval FDG-PET to death | 1.000 | 0.000 | 1.000 | 0.000 | 1.000 | -0.013 | 0.010 | -0.031 | 0.005 |
| TDP-43 pathology, binarized | 0.500 | 0.500 | 0.301 | 0.699 | 0.430 | 0.004 | 0.022 | -0.040 | 0.059 |

P(incl) – prior probability of inclusion. P(excl) – prior probability of exclusion. P(incl|data) – posterior probability of inclusion. P(excl|data) – posterior probability of exclusion. BF_inclusion_ – BF in favor of including the effect.

**Supplementary table 5.**

Bayesian regression analysis – posterior summaries of coefficients in the models predicting similarity to the limbic-predominant subtype in a subsample (n = 49).

| Coefficient | P(incl) | P(excl) | P(incl\|data) | P(excl\|data) | BF_inclusion_ | Mean | SD | 95% Credible Interval | |
| --- | --- | --- | --- | --- | --- | --- | --- | --- | --- |
|  |  |  |  |  |  |  |  | Lower | Upper |
| Intercept | 1.000 | 0.000 | 1.000 | 0.000 | 1.000 | 1.023 | 0.026 | 0.973 | 1.071 |
| Age | 1.000 | 0.000 | 1.000 | 0.000 | 1.000 | 0.002 | 0.003 | -0.004 | 0.008 |
| Sex | 1.000 | 0.000 | 1.000 | 0.000 | 1.000 | 0.018 | 0.053 | -0.085 | 0.118 |
| Interval FDG-PET to death | 1.000 | 0.000 | 1.000 | 0.000 | 1.000 | -0.029 | 0.013 | -0.055 | -0.006 |
| CA1 NFT density | 0.500 | 0.500 | 0.309 | 0.691 | 0.447 | 0.000 | 0.014 | -0.043 | 0.022 |

P(incl) – prior probability of inclusion. P(excl) – prior probability of exclusion. P(incl|data) – posterior probability of inclusion. P(excl|data) – posterior probability of exclusion. BF_inclusion_ – BF in favor of including the effect.

**Supplementary table 6.**

Bayesian regression analysis – comparison of models predicting similarity to the limbic-predominant subtype, in a subsample of participants with ADNC score of 1 and higher.

|  | P(M) | P(M\|Data) | BF_M_ | BF_10_ | R^2^ |
| --- | --- | --- | --- | --- | --- |
| Null model (including age, sex, interval FDG-PET to death) | 0.143 | 0.029 | 0.177 | 1.000 | 0.115 |
| Thal phase | 0.024 | 0.009 | 0.370 | 1.875 | 0.164 |
| Braak stage | 0.024 | 0.007 | 0.274 | 1.390 | 0.155 |
| TDP-43 pathology | 0.024 | 0.003 | 0.109 | 0.555 | 0.125 |
| Hippocampal sclerosis | 0.024 | 0.017 | 0.688 | 3.462 | 0.183 |
| Lewy body pathology | 0.024 | 0.099 | 4.522 | 20.829 | 0.235 |
| CERAD neuritic plaque score | 0.024 | 0.004 | 0.170 | 0.867 | 0.139 |

Rows with variables represent separate models in which only the specified variable is included; each model is compared to the null model with age, sex and interval between FDG-PET and death. P(M) – prior distribution, P(M|Data) – posterior distribution, BF_M_ represents how informative data is given prior and posterior distributions, BF_10_ – BF in favor of the respective model vs the null model.

**Supplementary table 7.**

Bayesian regression analysis – posterior summaries of coefficients in the models predicting similarity to the limbic-predominant subtype, in a subsample of participants with ADNC score of 1 and higher.

| Coefficient | P(incl) | P(excl) | P(incl\|data) | P(excl\|data) | BF_inclusion_ | Mean | SD | 95% Credible Interval | |
| --- | --- | --- | --- | --- | --- | --- | --- | --- | --- |
|  |  |  |  |  |  |  |  | Lower | Upper |
| Intercept | 1.000 | 0.000 | 1.000 | 0.000 | 1.000 | 1.030 | 0.020 | 0.996 | 1.074 |
| Age | 1.000 | 0.000 | 1.000 | 0.000 | 1.000 | 0.004 | 0.003 | 0.000 | 0.010 |
| Sex | 1.000 | 0.000 | 1.000 | 0.000 | 1.000 | 0.029 | 0.046 | -0.058 | 0.116 |
| Interval FDG-PET to death | 1.000 | 0.000 | 1.000 | 0.000 | 1.000 | -0.014 | 0.009 | -0.030 | 0.005 |
| Thal phase | 0.500 | 0.500 | 0.444 | 0.556 | 0.798 | -0.008 | 0.017 | -0.051 | 0.013 |
| Braak stage | 0.500 | 0.500 | 0.396 | 0.604 | 0.655 | -0.002 | 0.013 | -0.031 | 0.025 |
| TDP-43 pathology | 0.500 | 0.500 | 0.365 | 0.635 | 0.574 | 0.001 | 0.008 | -0.022 | 0.020 |
| Hippocampal sclerosis | 0.500 | 0.500 | 0.590 | 0.410 | 1.436 | 0.064 | 0.076 | -0.010 | 0.225 |
| Lewy body pathology | 0.500 | 0.500 | 0.878 | 0.122 | 7.166 | -0.086 | 0.049 | -0.158 | 0.000 |
| CERAD neuritic plaque score | 0.500 | 0.500 | 0.396 | 0.604 | 0.654 | -0.004 | 0.017 | -0.047 | 0.026 |

P(incl) – prior probability of inclusion. P(excl) – prior probability of exclusion. P(incl|data) – posterior probability of inclusion. P(excl|data) – posterior probability of exclusion. BF_inclusion_ – BF in favor of including the effect.

**Supplementary table 8.**

Bayesian ANCOVA – model averaged posterior summary in the analysis comparing similarity to the limbic-predominant subtype across pathology groups split by AD and TDP-43 pathology.

| Variable | Group | Mean | SD | 95% Credible Interval | |
| --- | --- | --- | --- | --- | --- |
|  |  |  |  | Lower | Upper |
| Intercept |  | 1.039 | 0.022 | 0.995 | 1.082 |
| Pathology group | No AD, no TDP-43 | -0.080 | 0.041 | -0.165 | -0.001 |
|  | AD only | 0.018 | 0.029 | -0.041 | 0.074 |
|  | TDP-43 only | 0.113 | 0.047 | 0.023 | 0.207 |
|  | AD and TDP-43 | -0.050 | 0.031 | -0.114 | 0.010 |
| Age |  | 0.005 | 0.003 | 0.000 | 0.010 |
| Sex |  | 0.044 | 0.041 | -0.040 | 0.127 |
| Interval FDG-PET to death |  | -0.011 | 0.009 | -0.030 | 0.007 |

**Supplementary table 9.**

Bayesian ANCOVA – comparison of models predicting similarity to the limbic-predominant subtype across pathology groups split by AD and LB pathology.

|  | P(M) | P(M\|Data) | BF_M_ | BF_10_ | Error % |
| --- | --- | --- | --- | --- | --- |
| Null model (including age, sex, interval FDG-PET to death) | 0.500 | 0.285 | 0.398 | 1.000 |  |
| Pathology group, split by AD and LB | 0.500 | 0.715 | 2.513 | 2.513 | 0.518 |

P(M) – prior distribution, P(M|Data) – posterior distribution, BFM represents how informative data is given prior and posterior distributions, BF_10_ – BF in favor of the respective model vs the null model. Error % represents numerical error of BF.

**Supplementary table 10.**

Bayesian ANCOVA – post-hoc t-tests in the analysis comparing similarity to the limbic-predominant subtype across pathology groups split by AD and LB pathology.

| Group 1 | Group 2 | Prior Odds | Posterior Odds | BF10, uncorrected | Error % |
| --- | --- | --- | --- | --- | --- |
|  |  |  |  |  |  |
| No AD, no LB | AD only | 0.414 | 0.285 | 0.688 | 0.003 |
| No AD, no LB | LB only | 0.414 | 0.915 | 2.208 | 0.005 |
| No AD, no LB | AD and LB | 0.414 | 5.057 | 12.208 | 0.000 |
| AD only | LB only | 0.414 | 0.293 | 0.708 | 0.003 |
| AD only | AD and LB | 0.414 | 0.442 | 1.066 | 0.008 |
| LB only | AD and LB | 0.414 | 0.147 | 0.354 | 0.003 |

BF_10_, uncorrected – BF in favor of the alternative hypothesis that values differ. Error % represents numerical error of BF.

**Supplementary table 11.**

Bayesian ANCOVA – model averaged posterior summary in the analysis comparing similarity to the limbic-predominant subtype across pathology groups split by AD and LB pathology.

| Variable | Group | Mean | SD | 95% Credible Interval | |
| --- | --- | --- | --- | --- | --- |
|  |  |  |  | Lower | Upper |
| Intercept |  | 1.036 | 0.022 | 0.991 | 1.079 |
| Pathology group | No AD, no LB | 0.080 | 0.042 | 0.001 | 0.168 |
|  | AD only | 0.026 | 0.031 | -0.038 | 0.088 |
|  | LB only | -0.065 | 0.042 | -0.153 | 0.013 |
|  | AD and LB | -0.041 | 0.030 | -0.102 | 0.015 |
| Age |  | 0.005 | 0.003 | 0.000 | 0.011 |
| Sex |  | 0.011 | 0.043 | -0.074 | 0.098 |
| Interval FDG-PET to death |  | -0.013 | 0.009 | -0.032 | 0.006 |

**Supplementary table 12.**

Bayesian ANCOVA – comparison of similarity to the limbic-predominant subtype between a group with no LB pathology and a group with limbic or amygdala-predominant LB.

|  | P(M) | P(M\|Data) | BF_M_ | BF_10_ | Error % |
| --- | --- | --- | --- | --- | --- |
| Null model (including age, sex, interval FDG-PET to death) | 0.500 | 0.269 | 0.369 | 1.000 |  |
| Limbic or amygdala-predominant LB | 0.500 | 0.731 | 2.711 | 2.711 | 1.334 |

P(M) – prior distribution, P(M|Data) – posterior distribution, BF_M_ represents how informative data is given prior and posterior distributions, BF_10_ – BF in favor of the respective model vs the null model. Error % represents numerical error of BF.

**Supplementary table 13.**

Bayesian ANCOVA – model averaged posterior summary in the analysis comparing similarity to the limbic-predominant subtype between a group with no LB pathology and a group with limbic or amygdala-predominant LB.

| Variable | Group | Mean | SD | 95% Credible Interval | |
| --- | --- | --- | --- | --- | --- |
|  |  |  |  | Lower | Upper |
| Intercept |  | 1.042 | 0.027 | 0.987 | 1.095 |
| Pathology group | No LB | 0.053 | 0.027 | 0.001 | 0.106 |
|  | Limbic or amygdala-predominant LB | -0.053 | 0.027 | -0.107 | -0.001 |
| Age |  | 0.006 | 0.003 | 0.000 | 0.012 |
| Sex |  | 0.018 | 0.048 | -0.076 | 0.117 |
| Interval FDG-PET to death |  | 0.000 | 0.011 | -0.023 | 0.021 |

**Supplementary table 14.**

Bayesian ANCOVA – comparison of similarity to the limbic-predominant subtype between a group with no LB pathology and a group with neocortical LB pathology.

|  | P(M) | P(M\|Data) | BF_M_ | BF_10_ | Error % |
| --- | --- | --- | --- | --- | --- |
| Null model (including age, sex, interval FDG-PET to death) | 0.500 | 0.059 | 0.062 | 1.000 |  |
| Neocortical LB pathology | 0.500 | 0.941 | 16.025 | 16.025 | 0.955 |

P(M) – prior distribution, P(M|Data) – posterior distribution, BFM represents how informative data is given prior and posterior distributions, BF10 – BF in favor of the respective model vs the null model. Error % represents numerical error of BF.

**Supplementary table 15.**

Bayesian ANCOVA – model averaged posterior summary in the analysis comparing similarity to the limbic-predominant subtype between a group with no LB pathology and a group with neocortical LB pathology.

| Variable | Group | Mean | SD | 95% Credible Interval | |
| --- | --- | --- | --- | --- | --- |
|  |  |  |  | Lower | Upper |
| Intercept |  | 1.017 | 0.027 | 0.961 | 1.072 |
| Pathology group | No LB | 0.076 | 0.028 | 0.020 | 0.132 |
|  | Neocortical LB | -0.076 | 0.028 | -0.133 | -0.021 |
| Age |  | 0.006 | 0.003 | -0.001 | 0.013 |
| Sex |  | -0.017 | 0.051 | -0.121 | 0.082 |
| Interval FDG-PET to death |  | -0.011 | 0.012 | -0.036 | 0.014 |


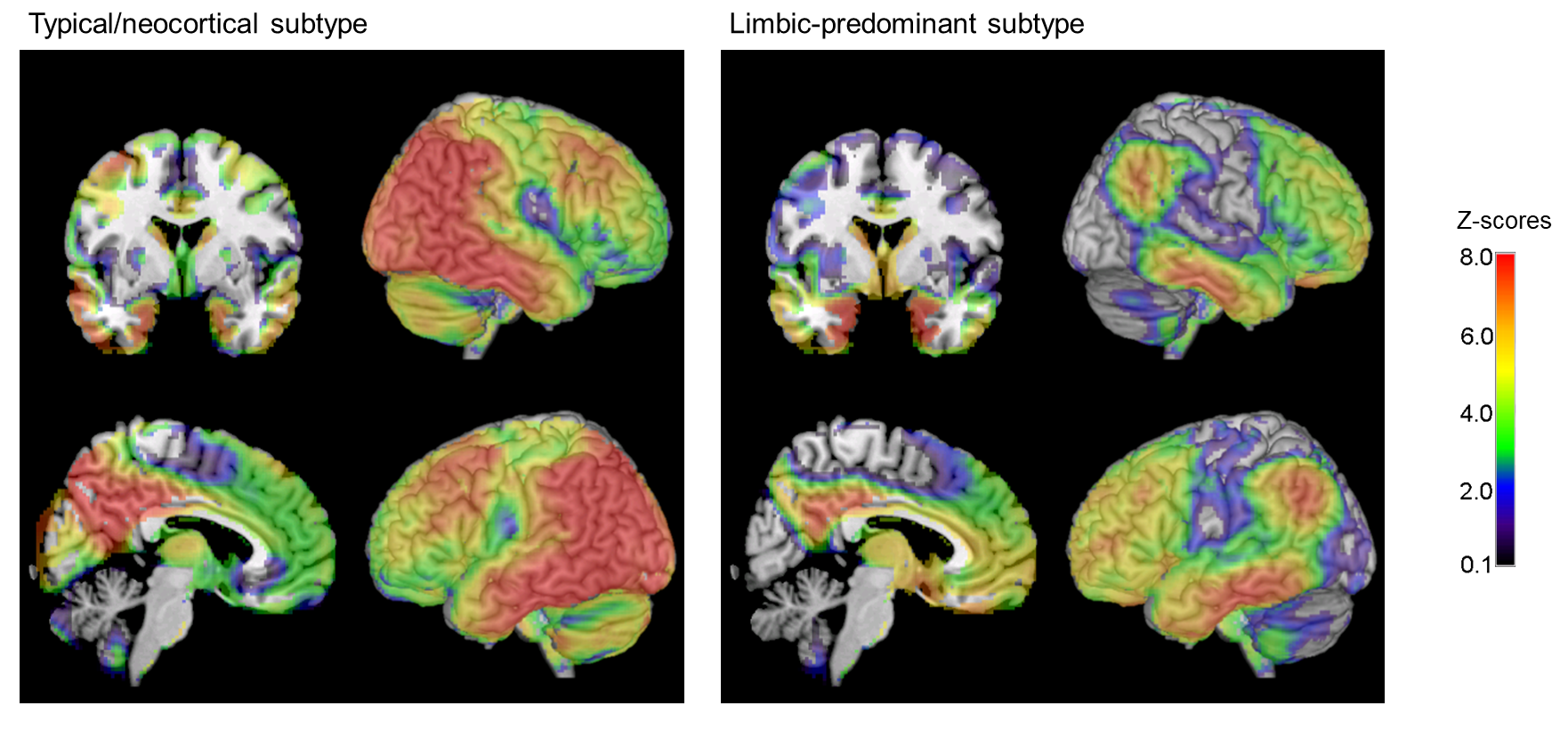


**Supplementary figure 1. Hypometabolic FDG-PET patterns of subtypes.**

Voxel-wise hypometabolic patterns of the typical/neocortical (n = 75) and the limbic-predominant (n = 69) subtypes were compared to a cognitively normal control group (n = 179). FDG-PET scans were adjusted to the average pons signal. Age, sex and years of education were used as covariates. Statistical maps of the group differences were converted into Z-scores.


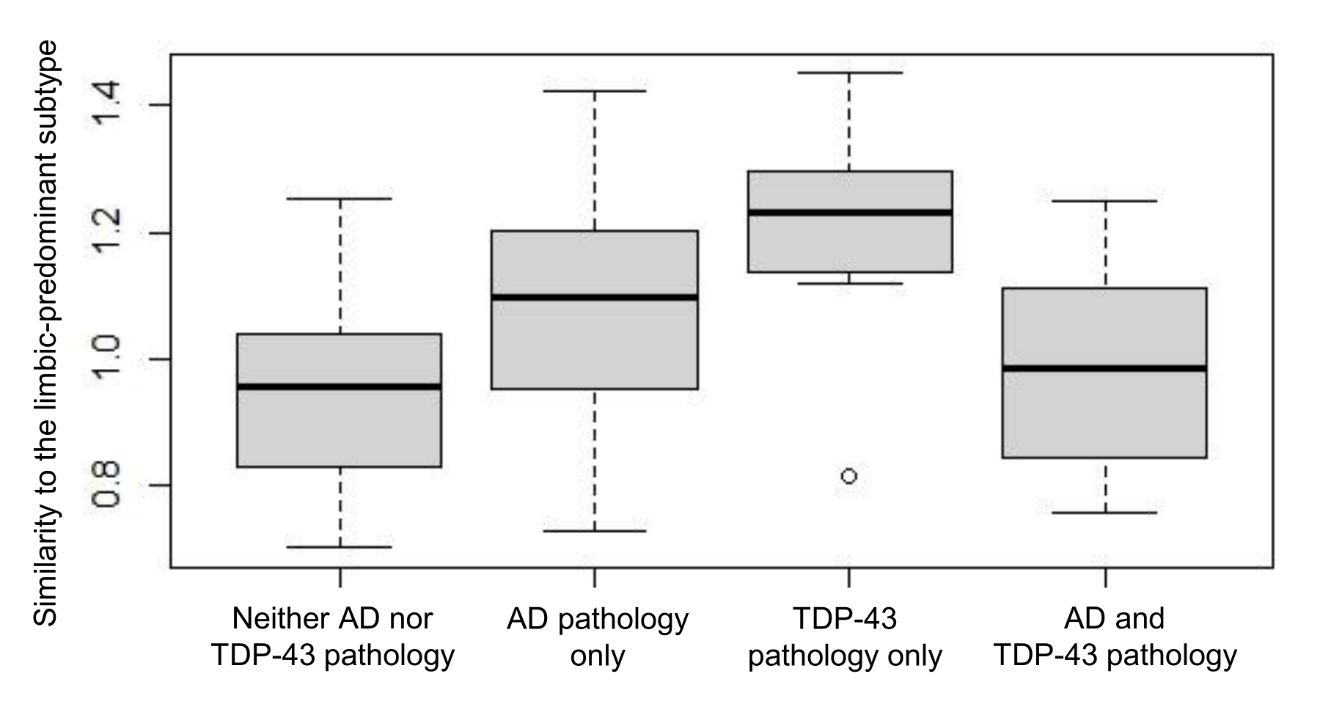


**Supplementary figure 2. Similarity to the limbic-predominant subtype across pathology groups split by AD and TDP-43 pathology.**

AD pathology is defined as ADNC score ≥ 2. TDP-43 pathology is defined as any TDP-43 inclusions identified in the assessed brain regions.


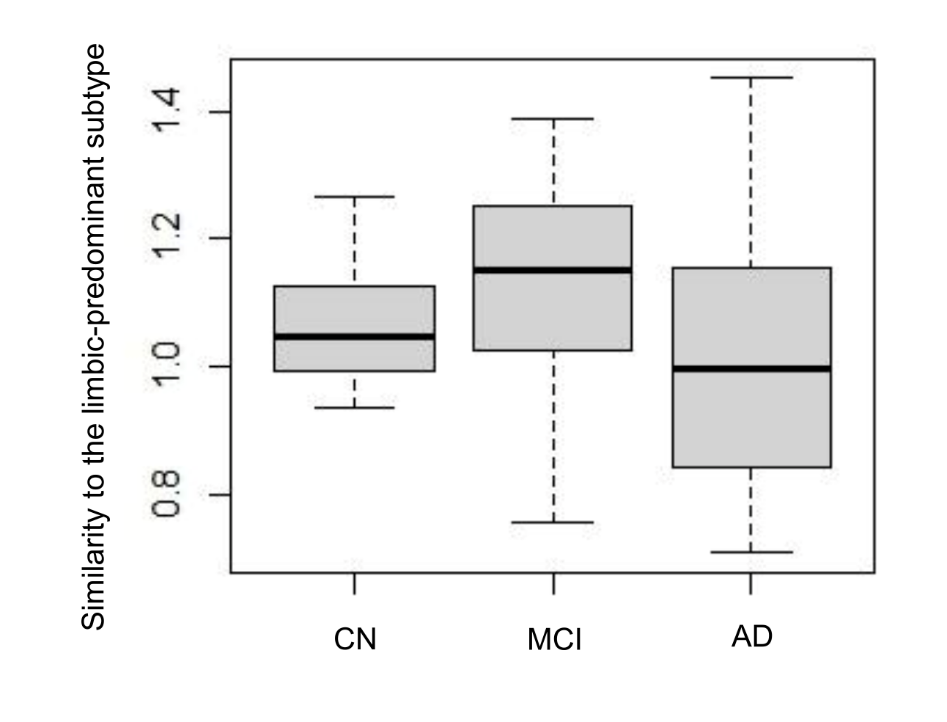


**Supplementary figure 3. Similarity to the limbic-predominant subtype across pathology groups split by diagnosis.**

Diagnoses are determined according to the last available clinical assessment.


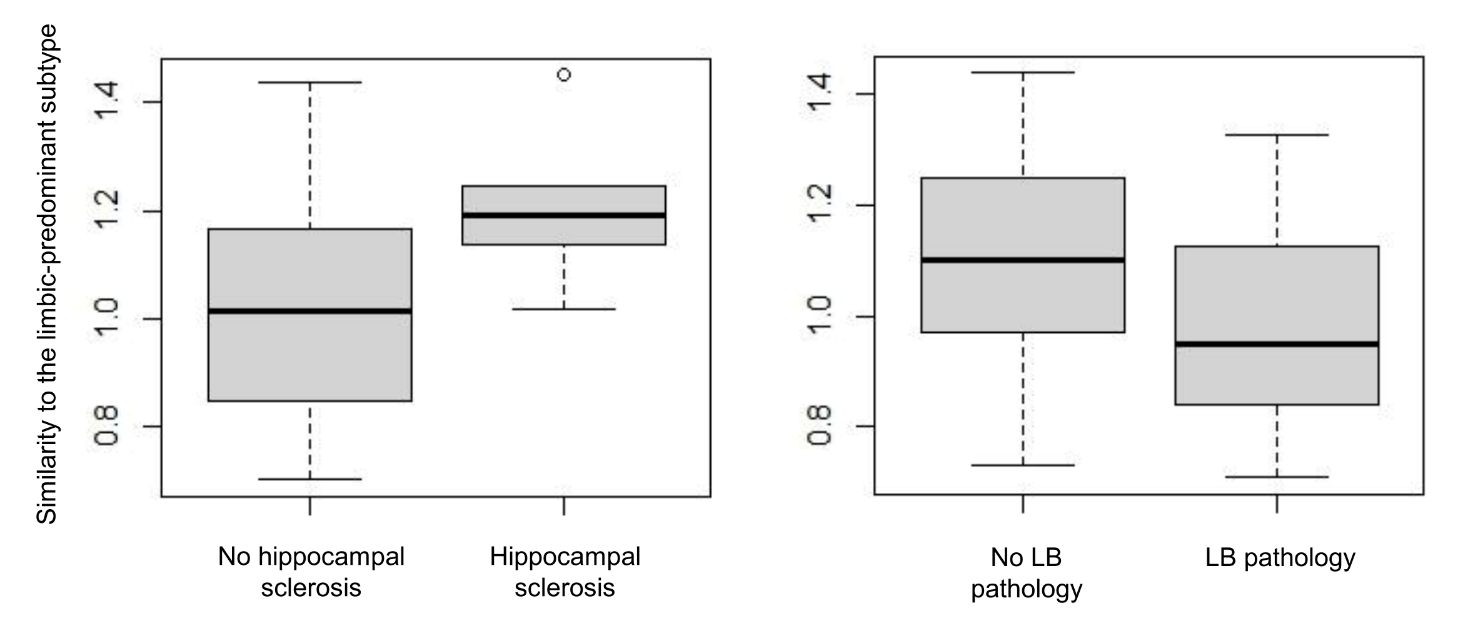


**Supplementary figure 4. Similarity to the limbic-predominant subtype across pathology groups split by presence of hippocampal sclerosis (left) and by presence of LB pathology (right).**
